# Recent insights into the structure and function of coronavirus ribonucleases

**DOI:** 10.1002/2211-5463.13414

**Published:** 2022-04-29

**Authors:** Meredith N. Frazier, Amanda A. Riccio, Isha M. Wilson, William C. Copeland, Robin E. Stanley

**Affiliations:** ^1^ Signal Transduction Laboratory Department of Health and Human Services National Institute of Environmental Health Sciences National Institutes of Health Research Triangle Park NC USA; ^2^ Genome Integrity and Structural Biology Laboratory Department of Health and Human Services National Institute of Environmental Health Sciences National Institutes of Health Research Triangle Park NC USA

**Keywords:** coronaviruses, drug design, endonucleases, exonucleases, SARS‐CoV‐2, structural biology

## Abstract

Coronaviruses use approximately two‐thirds of their 30‐kb genomes to encode nonstructural proteins (nsps) with diverse functions that assist in viral replication and transcription, and evasion of the host immune response. The SARS‐CoV‐2 pandemic has led to renewed interest in the molecular mechanisms used by coronaviruses to infect cells and replicate. Among the 16 Nsps involved in replication and transcription, coronaviruses encode two ribonucleases that process the viral RNA—an exonuclease (Nsp14) and an endonuclease (Nsp15). In this review, we discuss recent structural and biochemical studies of these nucleases and the implications for drug discovery.

## Graphical abstract

Coronaviruses encode two ribonucleases: Nsp14, an exonuclease, and Nsp15, an endonuclease. This review covers the latest structures of SARS‐CoV‐2 Nsp14 and Nsp15 and discusses insights into nuclease functions and progress towards therapeutic inhibitors. The viral particle was generated using BioRender.

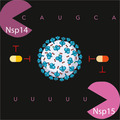

**Abbreviations**
ACE2: angiotensin‐converting enzyme 2
COVID‐19: coronavirus disease 2019
CoVs: coronaviruses
Cryo‐EM: cryo‐electron microscopy
DMVs: double‐membrane vesicles
dsRNA: double‐stranded RNA
EndoU: uridine‐specific endoribonuclease
ExoN: N‐terminal exonuclease domain
HCoV‐229E: human CoV‐229E
HTS: high‐throughput screening
IFN1: interferon 1
IFN‐β: beta interferon
MDA5: melanoma differentiation‐associated protein 5
MERS: Middle East respiratory syndrome
MHV: mouse hepatitis virus
MTase: methyltransferase
nsps: nonstructural proteins
ORF: open reading frame
PAMPs: pathogen‐associated molecular patterns
PEDV: porcine endemic diarrhea coronavirus
PRRs: pathogen recognition receptors
RdRp: RNA‐dependent RNA polymerase
RLRs: retinoic acid‐inducible gene‐I‐like receptors
RTC: replication–transcription complex
SAM: *S*‐adenosyl‐l‐methionine
SAMDI: self‐assembled monolayers for matrix‐assisted laser desorption ionization
SARS: severe acute respiratory syndrome
ssRNA: single‐stranded RNA
TFN: tumor necrosis factor
ZnF: zinc finger

## Overview of coronavirus life cycle and the nonstructural proteins

The Coronaviridae family is composed of enveloped, positive‐sense single‐stranded (ss) RNA viruses with large RNA genomes around 30 kb in size [1]. Coronaviruses (CoVs) are a significant public health and economic concern because they can infect humans and livestock, and other mammals and birds [2]. There are four genera of CoVs, which are designated Alpha, Beta, Gamma, and Delta, with Alpha and Beta primarily being responsible for infecting humans and other mammals. In addition to infecting mammals, Gamma and Delta CoVs also infect birds. CoVs that infect humans typically cause respiratory infections, ranging from mild to severe. Members of the Beta‐CoV family have caused several deadly outbreaks including the 2003 Severe Acute Respiratory Syndrome (SARS) outbreak, the 2012 Middle East Respiratory Syndrome (MERS) outbreak, and the ongoing SARS‐CoV‐2 outbreak [3, 4]. SARS‐CoV‐2 is the causative pathogen of the coronavirus disease 2019 (COVID‐19) pandemic that has impacted millions worldwide.

**Fig. 1:**
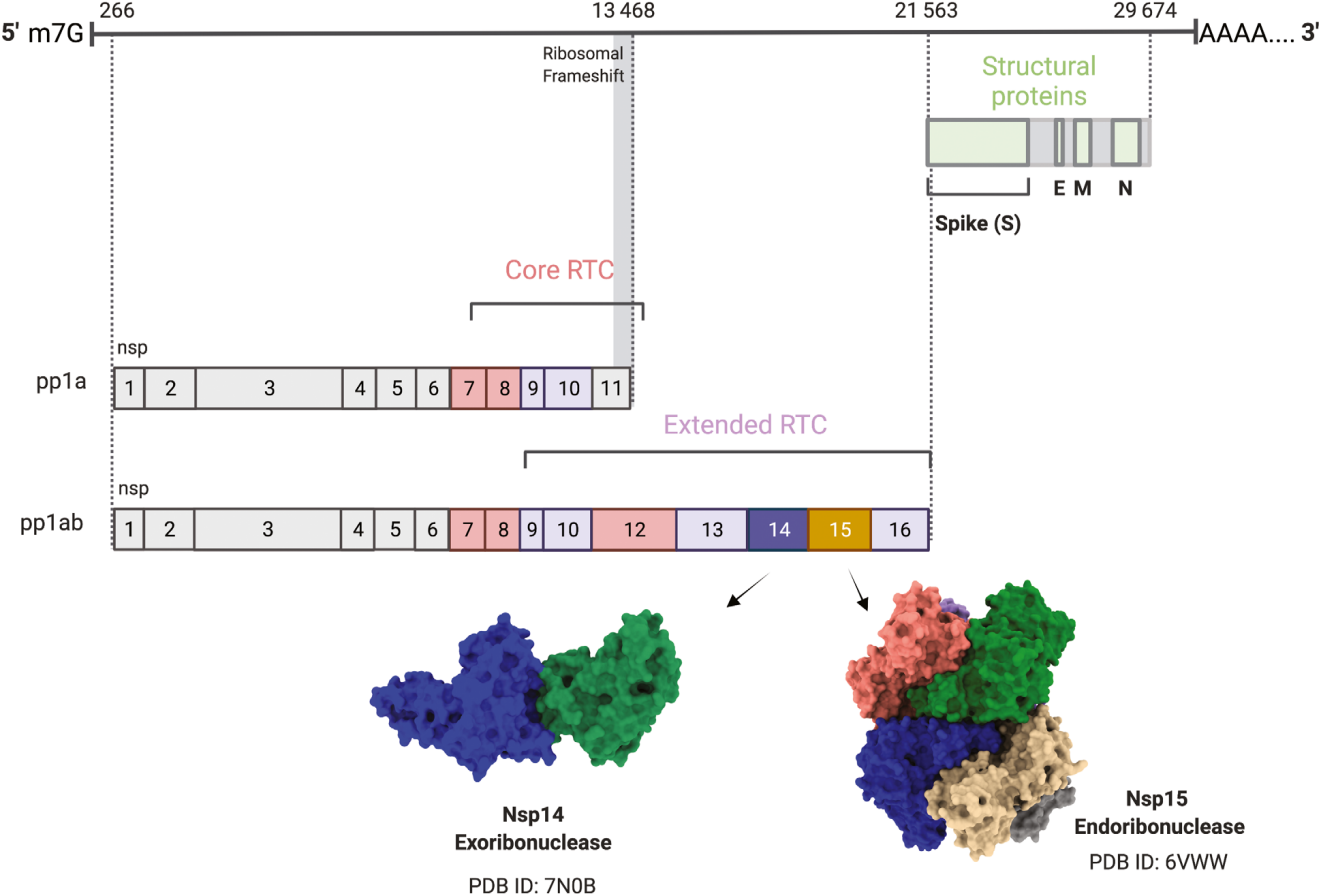
Overview of SARS‐CoV‐2 proteins. SARS‐CoV‐2 is a 5′ capped, 3′ polyadenylated single‐stranded, positive‐sense RNA virus. Two‐thirds of the genome encodes two open reading frames (generated by a ribosomal frameshift), which produce long polyproteins that are subsequently cleaved by viral proteases. Core members of the RTC are colored in light red, extended RTC members in purple. The final one‐third of the genome encodes structural and accessory proteins (structural proteins highlighted in green). The two nucleases are positioned beside each other in the genome, in orf1ab. Nsp14, the exoribonuclease, is followed by Nsp15, the endoribonuclease. Representative structures of the two nucleases are shown beneath the genome diagram. This image was generated using BioRender.

To successfully replicate the viral genome within the host, CoVs rely on multiple enzymes to replicate and process the viral RNA (reviewed in Refs [5, 6, 7]). The CoV infection cycle initiates with the attachment of the virus to a host receptor. For SARS‐CoV‐2, this is mediated via the spike protein, which coats the surface of the virus and the host receptor angiotensin‐converting enzyme 2 (ACE2) [8]. Following the entry into the cell and uncoating of the viral particle, the CoV ssRNA genome, which contains a 5′ cap and poly‐A tail, is released into the cytoplasm. The first two‐thirds of the viral genome encodes for large open reading frames (ORF1a and ORF1ab) that are translated by host ribosomes into two polypeptides known as pp1a and pp1ab [1]. Translation of pp1ab relies on a programmed ribosomal frameshift within the open reading frame [9]. The two polypeptides are then proteolyzed by viral encoded proteases into the nonstructural proteins (Nsp1‐16, Fig. 1), many of which assemble into a large replication‐transcription complex (RTC) [5]. The expression of ORF1a is higher than ORF1ab resulting in greater levels of Nsp1‐10 relative to the other nsps [10].

The RTC localizes to double‐membrane vesicles (DMVs), also known as replication organelles, that form following expression of the Nsps within the host [11, 12, 13]. The DMVs are thought to provide a protective environment for replication and transcription of the viral RNA. The first task of the RTC is to synthesize the full‐length negative strand, which serves as the template to generate copies of the genomic RNA and the subgenomic mRNAs, which encode the structural viral proteins. Viral RNA synthesis is driven by the RTC‐core, which includes the RNA‐dependent RNA polymerase (RdRp, Nsp12) and its two cofactors Nsp7 and Nsp8 [5, 14]. Additional accessory members of the RTC include an RNA helicase/triphosphatase (Nsp13) thought to be important for RdRp backtracking [15], two methyltransferases (Nsp14 and Nsp16) and their cofactor (Nsp10), which are important for RNA capping [16], and Nsp9, which binds ssRNA and is also thought to facilitate capping (Fig. 1) [17]. The RTC co‐transcriptionally facilitates capping of the viral RNA, which is a multistep process and is critical for maintaining the stability of the viral RNA and blocking the activation of the host immune response [16, 18].

The RTC also includes two ribonucleases, the exoribonuclease Nsp14, which is a bi‐functional enzyme containing both an N7 methyltransferase and exonuclease domain, and the endoribonuclease, Nsp15 (Fig. 1). It is perplexing that CoVs encode for two ribonucleases that can degrade viral RNA. The conservation of Nsp14 and Nsp15 across the Coronaviridae family suggests that both nucleases are important for the viral life cycle, but that their activity must be tightly regulated to avoid excess destruction of genomic RNA. Nsp14 directly associates with the core RTC members to facilitate both viral RNA capping and replication fidelity [19]. The function of Nsp15 within the RTC is much less clear; Nsp15 co‐localizes with the other RTC members and molecular modeling suggests that Nsp15 could function as a central scaffold for the entire RTC [20, 21, 22]. Nsp14 and Nsp15 have also been shown to be important for evasion of the host immune response specifically by blocking the accumulation of double‐stranded (ds) RNA intermediates that form during replication (recently reviewed in Refs [23, 24]). Nucleic acids, including the dsRNA replication intermediate and the negative‐strand poly‐U sequence in CoVs, form a class of pathogen‐associated molecular patterns (PAMPs), which stimulate the innate immune system. Pathogen recognition receptors (PRRs) recognize specific PAMPs and initiate interferon signaling, sending the cell into an antiviral state. Thus, the Nsp14 and Nsp15 nucleases are attractive therapeutic targets.

In response to the emergence of SARS‐CoV‐2, there has been an explosion of structural and functional studies that have begun to define the molecular mechanisms of RNA processing by the CoV ribonucleases. In this review, we focus on recent groundbreaking work illuminating the structure, function, and RNA substrates of Nsp14 and Nsp15. These studies provide the foundation for the rationale development of inhibitors for Nsp14 and Nsp15, and we further highlight recent work and challenges associated with developing antiviral nuclease inhibitors.

## Nsp14

**Fig. 2:**
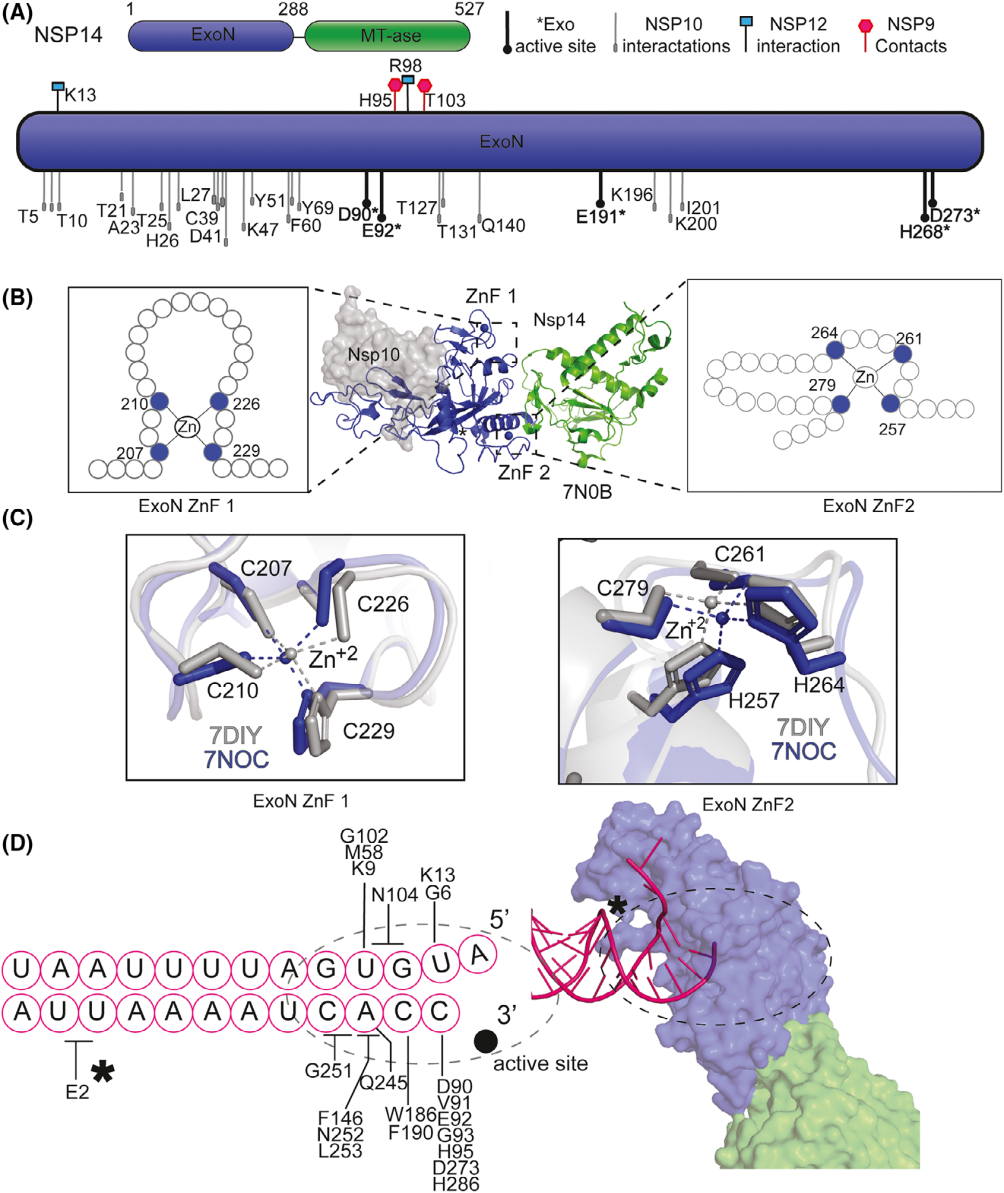
Nsp14 ExoN architecture. (A) (top, right) Overview schematic Nsp14 structure. Two domains are depicted: N‐terminal exonuclease domain (ExoN) (blue) and C‐terminal domain methyltransferase (MTase) (green). (top, left) Key for the protein–protein interaction schematic (below): exonuclease active sites (black and bold filled circle with an asterisk), Nsp10 interactions oval, gray‐filled, and black‐outlined, from [36, 44], Nsp12–Nsp14 interaction sites, black‐outlined cyan‐filled square, Nsp9‐Nsp14 magenta hexagon [19]. (bottom) ExoN residue numbering for either protein–protein interactions or active site residues (shown in bold and indicated with an asterisks). (B) (center) Two ZnF locations identified in dashed lines on PDB ID: 7N0B. Location of the zinc metals are in blue circles, and a gray surface representation is shown for the location of the NSP10 interaction. A cartoon of residues for ZnF1 (left) or ZnF2 (right). (C) Stick representation of ZnF1 (left) and ZnF2 (right) of PDB ID: 7DIY (gray) and 7N0C (blue) (Table 1). (D) Schematic of RNA used in 7N0B (pink circles) (left). Additional RNA that is unbound in original PDB was removed from image. Residues contacting the RNA base are indicated by a line; if a residue contacts more than one base, it is indicated by a crossbar. The active site is highlighted by a filled black circle. The asterisk and the dashed circle correspond to the same locations on the surface presentation of the structure (right). Surface cartoon representation of PDB ID: 7N0B. RNA (pink, cartoon), ExoN domain (blue surface), and MTase (green surface).

Nsp14 consists of an N‐terminal exonuclease domain (ExoN) coupled to a C‐terminal N7‐methyltransferase (MTase) domain (Fig. 2A). These two distinct domains are separated by a flexible hinge region composed of a 14‐residue loop (residues 286–299), which is thought to permit the independent movement of the two separate domains. Nsp14 is well‐conserved across the Coronaviridae family, and the ExoN domain is also found in many large nidoviruses suggesting that this exonuclease activity is important for large ssRNA viruses [25]. The C‐terminal MTase domain of Nsp14, in the presence of SAM (*S*‐adenosyl‐l‐methionine), methylates guanosine at the N7 position and forms m7GpppA, also known as a “cap‐0”. The capping process is critically important for the virus to escape innate immunity but is not the focus of this review (for recent reviews on the Nsp14 MTase domain please see [5, 16, 26]). Instead, the following sections will focus on recent work exploring the structure and function of the Nsp14 ExoN domain.

### Proofreading role in viral replication and immune response

The Nsp14 ExoN domain plays a critical role in viral replication, which is an inherently error‐prone process for RNA viruses. The error rate of replication for CoVs is estimated to be between 1/34 000 and 1/135 000 base pairs [27, 28]. This inaccurate mechanism of replication allows for the virus to adapt to the host environment. However, unresolved mismatches or misincorporations can lead to replication complex stalling and ultimately a lack of viral viability. The direct link of error‐prone replication and viability underpins the importance of proofreading. Nsp14 is responsible for proofreading and maintaining the integrity of the viral genome [29]. SARS‐CoV‐2 Nsp14 is a 527 amino acid protein and performs the proofreading function via the ExoN domain. Unlike SARS‐CoV‐1, mutations to SARS‐CoV‐2 Nsp14 ExoN, specifically Zinc Finger (ZnF)1 mutations, have been shown to eliminate viral viability and decrease the antiviral response [30]. In addition to the essential role of Nsp14 exonuclease activity for SARS‐CoV‐2 replication, the ExoN domain plays a key role in the evasion of host immune responses. Previous studies have indicated that Nsp14 is a beta interferon (IFN‐β) antagonist, but it was unclear whether this was ExoN‐driven. For both alpha‐CoV models and MHV (mouse hepatitis virus), a beta‐CoV‐like SARS‐CoV‐2, there is a support that it is in fact the ExoN, which blocks the activation of dsRNA sensors, such as expression of beta interferon IFN‐β and tumor necrosis factor (TFN) [31]. Mutant ExoN in MHV reduces viability, while this same mutation in alpha‐CoV models is lethal. Furthermore, viruses lacking ExoN activity are more sensitive to interferon signaling pretreatment than wild‐type (WT) [32]. In total, the critical roles that Nsp14 plays in proofreading, capping, and modulating the innate immune system make it an ideal target for structural studies and inhibitor design.

### Structural overview of the exonuclease domain

The ExoN domain performs 3 overarching functions: (a) metal‐binding‐dependent exonuclease activity, (b) supporting protein–protein interactions, and (c) RNA binding. Nsp14 belongs to the DEDDh/DEEDh exonuclease family, which performs nucleic acid exonuclease activity in a 3′–5′ direction. Other members of this superfamily include *Escherichia coli* DNA polymerase III ɛ subunit and *E. coli* RNase T [33]. Although it had been predicted from homology models [26, 34], recently multiple high‐resolution structures have described the SARS‐CoV‐2 Nsp14 metal‐binding sites (Table 1). The active site is coordinated by residues: D90, E92, E191, D273, and H268 (Fig. 2A, additional discussion below).

**Table 1:** PDB IDs for Nsp14 SARS‐CoV‐2. PDBs of SARS‐CoV‐2 Nsp14‐containing coordinates are listed in order of acceptance publication date. Model residue numbering is indicated only for Nsp14. APO is defined as not containing nucleic acid. PDB accepted abbreviation are used for ethylene glycol (EDO), zinc (Zn), magnesium (Mg), and calcium (Ca), divalent charge (2+), and wild‐type (WT).

PDB ID	Protein components	Resolution (Å)	State (ligand)	Structural technique	Reference
7DIY	1‐294 Nsp14 (ExoN)/Nsp10	2.69	APO, Zn^2+^, Mg^2+^	X‐ray diffraction	[44]
7EGQ	3‐525 Nsp14/Nsp10/Nsp8/Nsp12/Nsp7/Nsp13/Nsp9	3.35	dsRNA not Nsp14 bound, Zn^2+^, Mg^2+^	Cryo‐EM	[19]
7EIZ	3‐525 Nsp14/Nsp10/Nsp8/Nsp12/Nsp7/Nsp13/Nsp9	3.8			
7N0B	3‐524 Nsp14 WT/Nsp10	3.9	dsRNA mismatch, Zn^2+,^ Ca^2+^	Cryo‐EM	[36]
7N0C	Nsp10‐Nsp14 E191A	3.4	dsRNA mismatch, Zn^2+,^ Ca^2+^		
7N0D	Nsp10‐Nsp14 E191A‐tetramer	2.5	dsRNA, mismatch Zn^2+^, Mg^2+^		
7MC5	3‐287 Nsp14 (ExoN)/Nsp10 E191Q	1.64	APO, Zn^2+^, EDO	X‐ray diffraction	[43]
7MC6	3‐289 Nsp14 (ExoN) E191Q/Nsp10	2.10	APO, Zn^2+^, Mg^2+^		

The ExoN domain is also critical for supporting protein–protein interactions between other RTC members. The most well‐characterized protein–protein interaction with Nsp14 ExoN is Nsp10. In the absence of Nsp10, Nsp14 exonuclease activity is negligible; however, the mechanism of stimulation is still unknown. Possible explanations include structural support or increased RNA‐binding contacts [35]. Cryo‐Electron Microscopy (EM) structures of the core RTC with multiple accessory factors revealed that the ExoN domain also makes contacts with additional RTC members including Nsp12, Nsp8, and Nsp9 (Fig. 2A) [19]. Liu et al. [36] have recently shown cryo‐EM density for an interaction surface between Nsp14 and Nsp8 with a mismatch dsRNA substrate; however, the density was not characterized, so residues that define this interface have not been resolved.

Thus far quantitative *in vitro* RNA binding has not been shown, but recent structural analysis displays the extensive interface between the ExoN of Nsp14 and mismatch RNA. The ExoN domain contains two ZnF motifs, described as ZnF1 and ZnF2, which contribute to protein stability and likely permit RNA binding [37, 38]. The amino acid spacing of ZnF1 more closely resembles a classical assembly of a Cys_4_ ZnF, C‐(X_2_)‐C‐(X_14_)‐C‐(X_2_)‐C; however, the spacing of the CCHC of the second ZnF has a long‐range nonclassical assembly, C‐(X_2_)‐C‐(X_3_)‐H‐(X_13_)‐C (Fig. 2B,C). Importantly, recent alignment and structural analysis described nearly identical positions for the ZnFs, regardless of ligand or substrate status (Fig. 2C) [36]. Despite the importance of ZnFs for the overall folding of Nsp14, structures bound to mismatch RNA also highlight that Nsp14 makes numerous contacts with the RNA outside of the ZnF “recognition‐helix” (Fig. 2D) [34].

### Nsp14 cleavage mechanism

As stated above the activity of Nsp14 *in vitro* is reliant on an accessory factor, Nsp10. Nsp10 enhances activity up to 260‐fold [35]. However, it seems that Nsp10 does not directly contribute to the cleavage mechanism of Nsp14, but rather either provides support for the active site and/or contributes to the stability of RNA within the active site. SARS‐CoV‐2 Nsp14 exonuclease activity is metal‐dependent and similarly to Nsp14 of SARS‐CoV‐1 uses Mg^2+^ over Mn^2+^, while Ca^2+^ and Zn^2+^ are not supported [36, 39]. Previous structural analyses have shown one occupied metal‐binding site for SARS‐CoV‐1 Nsp14; however, biochemical analysis of SARS‐CoV‐1 Nsp14, as measured by isothermal titration calorimetry, describes a two‐metal‐binding site [40]. Generally, one‐metal ion mechanisms tend to be less stringent and substrate specificity is less discriminant.

**Fig. 3:**
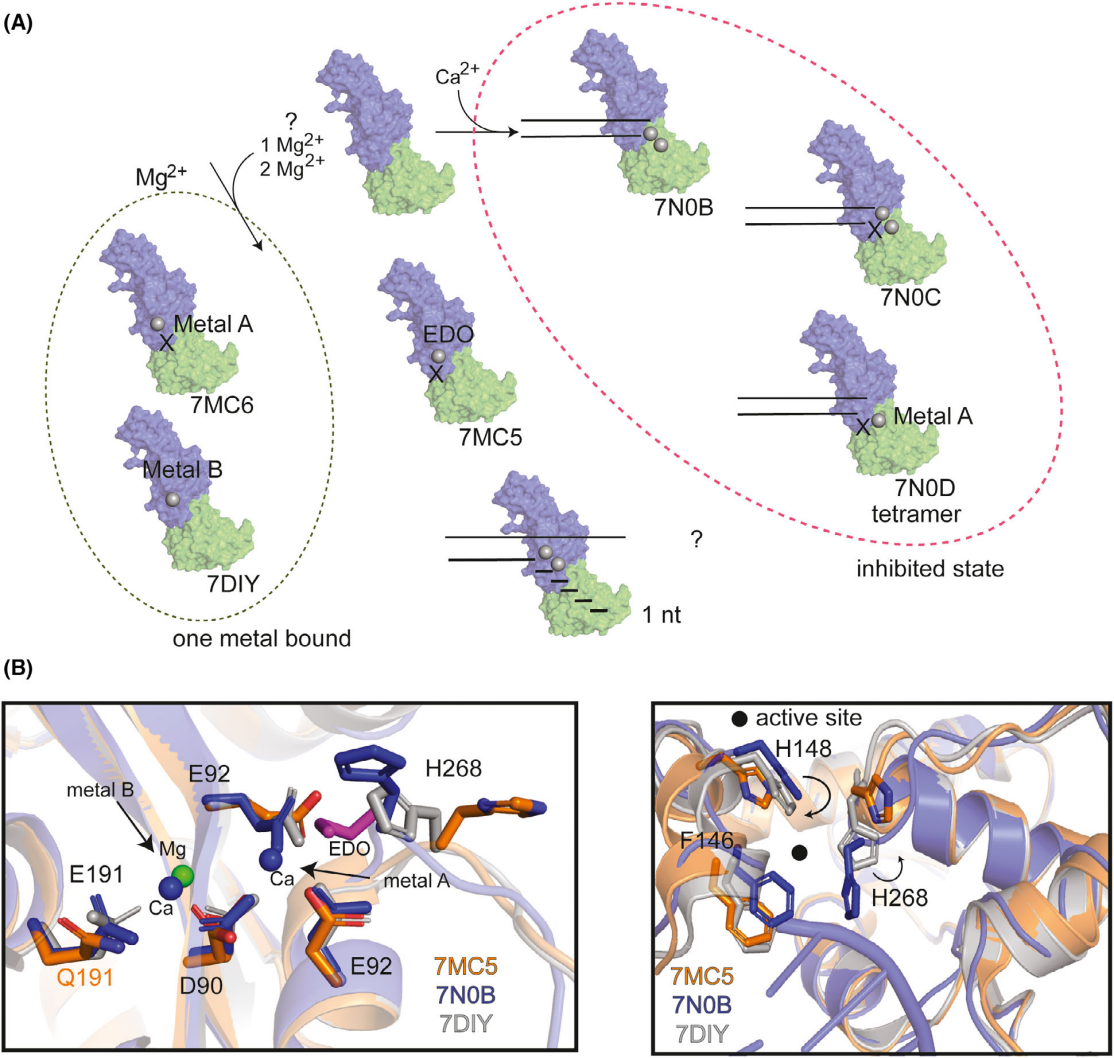
Nsp14 exonuclease cleavage mechanism. (A) Overview of current mechanistic analysis in structural studies with corresponding PDB IDs below the transparent cartoon surface representation of Nsp14. Dashed oval (green, left) structures only have one‐metal site bound with Mg^2+^. Dashed oval (magenta, right) indicates an inhibited (Ca^2+^‐bound states). Two parallel lines are a cartoon schematic of RNA. PDB ID: 7N0B was used to generate the surface presentation, which is colored by domain (ExoN, blue and MTase, green). Ethylene glycol (EDO). X indicates a mutated active site enzyme. (B) (left) Active site residues are shown in sticks and transparent cartoon presentation of the zoomed‐in exoN. Three PDBs were analyzed 7MC5 (orange), 7N0B (blue), and 7DIY (gray). 7MC5 has a E191Q mutation (orange text), all other residues written in black text and the EDO (purple) is shown in stick representation. Metal site locations are indicated with arrows. (right) Cartoon representation of PDBs globally aligned to 7DIY and movement is indicated by black arrows in the location of RNA binding. Select residues are highlighted by sticks and colored according to the PDB color. A filled black circle highlights the location of the active site.

Recently, SARS‐CoV‐2 Nsp14 structures have been detailed in various liganded states and varied metal site occupancies (Fig. 3A and Table 1). To date, substrate‐bound structures are occupied by Ca^2+^ in the active site and all in the presence of the cofactor, Nsp10 (Fig. 3A and Table 1, PDB ID 7N0B–7N0D, [36]). Despite the difference in ligand conditions and protein mutations, and biochemical and technique differences in high‐resolution X‐ray crystallography or cryo‐EM structures, the ExoN domain shares a similar position. One notable exception is the movement in the loop containing histidine 268 (H268) (Fig. 3B). In some instances, movement of this histidine could indicate participation in exonuclease activity. In a one‐metal activation mechanism, the histidine could act as a general base to deprotonate and activate the nucleophilic water, whereas in a two‐metal activation mechanism, metal A activates the water molecule and metal B promotes product exit.

The step‐by‐step reaction mechanism of Nsp14 exonuclease activity is still under investigation. Excision proceeds in single base increments as confirmed by sizing analysis performed by Baddock et al. [41], which indicates there is a nucleophilic attack of the scissile phosphodiester, and protonation of the leaving group, catalyzing a 3′–5′ nucleoside monophosphate exit. Likewise, a self‐assembled monolayer for matrix‐assisted laser desorption ionization (SAMDI) mass spectrometry assay indicates that Nsp14/Nsp10 requires a 3′OH, confirming that the enzyme acts in a 3′–5′ direction and that the first nucleotide likely is digested before proceeding with subsequent activities [42]. Comparing the structural coordinates of Nsp14 in metal‐free (7MC5, [43]), metal B site bound (7DIY, [44]), and substrate and metal A and B site bound (7N0B, [36]) states, reveals that the engagement of substrate and metals results in a conformational change surrounding the active site and the substrate‐binding site (Fig 3B, right). While the structural studies do not fully detail the mechanism of Nsp14 exonuclease activity, these recent structures provide the framework for future structural studies. It is worth noting that while Nsp10 does not directly contribute to the Nsp14 exonuclease enzymatic reaction, it plays a substantial role in the efficiency of Nsp14‐dependent RNA cleavage.

### Nsp14 substrate specificity

Nsp14 is active on many different RNA substrates. In SARS‐CoV‐1 it was shown *in vitro* and in the presence of Nsp10, that Nsp14 displays increased 3′–5′ exonuclease activity on a dsRNA substrate, less activity on an ssRNA substrate, and negligible activity on dsDNA [40, 45]. While recent structural analysis of SARS‐CoV‐2 revealed Nsp14/Nsp10 bound to dsRNA with a mismatch (Fig. 3A), the preference for dsRNA seems less apparent. For example, Ma et al. [46] have shown biochemically that Nsp10/14 hydrolyzes RNAs in dsRNA, ssRNA, and RNA DNA/hybrid. Likewise, cleavage is observed on a mismatch at the 3′ end of RNA, while longer flaps or gaps showed no strong preference in exonuclease activity [41]. At high concentrations Nsp14, in the absence of Nsp10, has also been shown to minimally act on dsRNA substrates. Due to the limit of detection, other substrates have not been exhaustively studied for isolated Nsp14 activity [35]. Interestingly, *in vitro* and structural studies seem to support the observation that a 3′ OH is critical for the cleavage of misincorporated RNA bases [36]. Additionally, Nsp14 in both SARS‐CoV‐1 and ‐2 show no sequence specificity [41]. These same analyses show that activity is substantially reduced on poly‐U or poly‐A RNA perhaps supported by the hypothesis that Nsp14 would have specificity for certain types of ternary structures in SARS‐COV‐2 [26].

## Nsp15

**Fig. 4:**
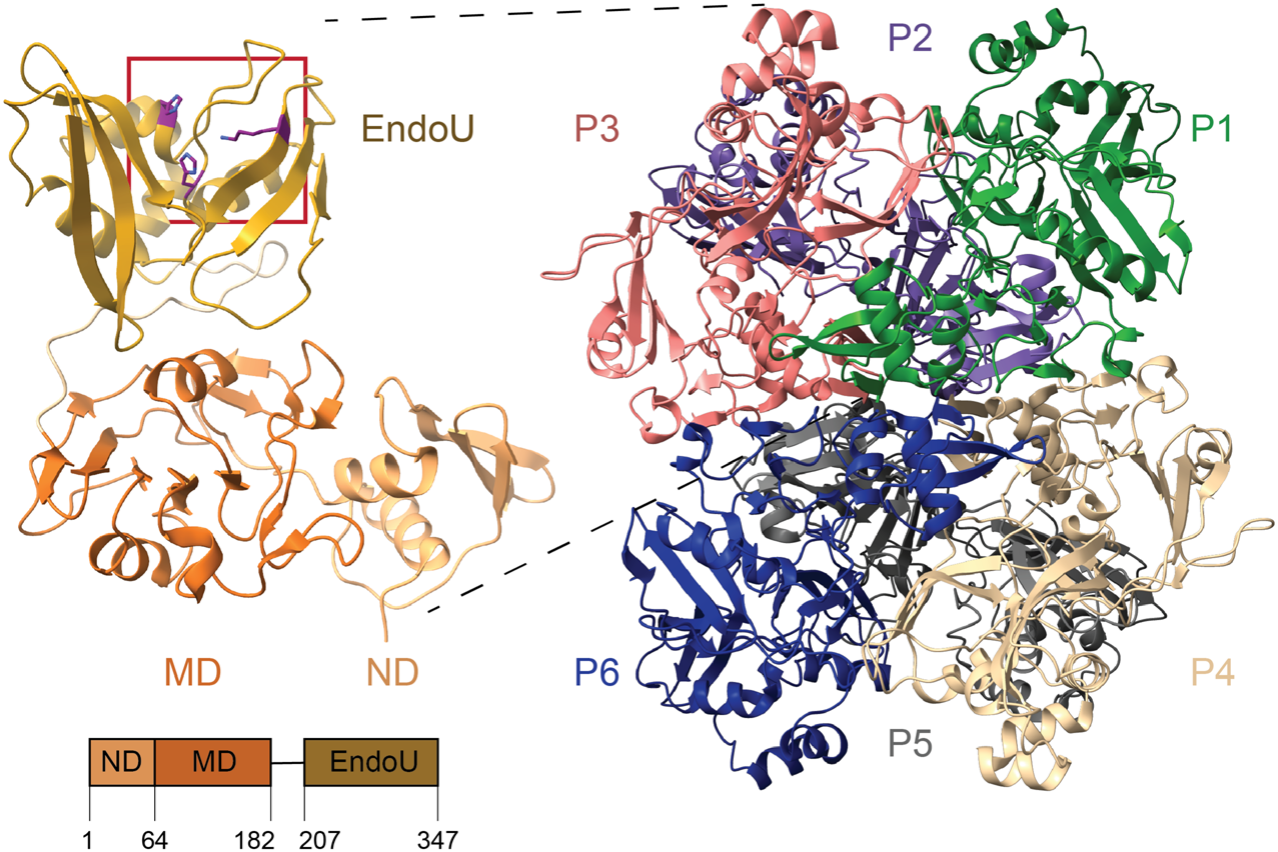
Nsp15 architecture. Left, one Nsp15 protomer is colored by domain: N‐terminal domain (ND), middle domain (MD), catalytic endoribonuclease domain (EndoU) with residue boundaries defined on a domain diagram below. The active site is boxed in red, and the catalytic triad residues (H235, H250, K290) are shown in stick format. Right, the Nsp15 hexamer is shown, and the protomers are labeled. PDB ID: 6VWW was used to model the apo Nsp15 structure.

Nsp15 is a uridine‐specific endoribonuclease comprised of three domains: N‐terminal, Middle, and the catalytic EndoU (uridine‐specific endoribonuclease) domain (Fig. 4). Oligomerization of Nsp15 is critical for enzyme function—monomeric Nsp15 has no nuclease activity [47]. Interactions across all three domains mediate oligomerization. In alpha‐ and beta‐CoVs, Nsp15 forms a hexamer, although *in vitro* analyses of a porcine delta‐CoV suggest hexamerization is not always conserved [48, 49, 50, 51, 52, 53, 54]. The EndoU domain is more broadly conserved across nidoviruses, suggesting this nuclease activity is critical for large, positive‐sense RNA viruses. In arteriviruses, for example, the EndoU domain resides in Nsp11 [54]. EndoU domains are also found in all kingdoms of life. In eukaryotes, EndoU domain‐containing proteins have cellular functions in processing snoRNAs [55]. Bacteria possess EndoU proteins that function in toxin‐antitoxin systems to respond to stress [56, 57, 58]. During the COVID‐19 pandemic, several new Nsp15 structures have been determined to capture the EndoU domain in different steps of the cleavage pathway, which has resulted in new insights into the mechanisms of cleavage and immune system evasion, and new considerations for drug design.

### Role in evasion of the immune response

Initially, Nsp15 was believed to play a direct role in viral replication [59, 60]. However, further work demonstrated that Nsp15 nuclease activity is not necessary for viral replication. Instead, Nsp15 nuclease activity is critical in the evasion of the host immune response to the virus, specifically by preventing the activation of viral RNA sensors [61, 62]. In this way, Nsp15 antagonism of the host immune system promotes viral replication without playing a direct role in replication. Nsp15 cleaves viral RNA to evade detection by the PRR melanoma differentiation‐associated protein 5 (MDA5), a member of the retinoic acid‐inducible gene‐I‐like receptors (RLRs) [61, 62]. Work with model animal CoVs has shown that viruses carrying mutated Nsp15—either destabilizing the protein or inactivating its enzyme activity—stimulates the MDA5‐dependent interferon (IFN) immune response cascade [61, 62]. Activation of MDA5 triggers the interferon 1 (IFN1) cascade (specifically, IFN‐⍺ and IFN‐β) [63]. In studies of porcine endemic diarrhea CoV (PEDV), viruses lacking active Nsp15 resulted in higher levels of type I responses in cells. Additionally, piglets infected with the Nsp15‐deficient virus had a much higher survival rate than those infected with virus‐containing WT Nsp15 [64]. Similar trends were seen with MHV, where mice immunized with Nsp15 nuclease deficient virus successfully cleared WT virus [61]. Studies with arterivirus Nsp11 have also shown that EndoU nuclease activity is important for the antagonism of type I interferons [65].

### Structural overview of the endonuclease domain

**Fig. 5:**
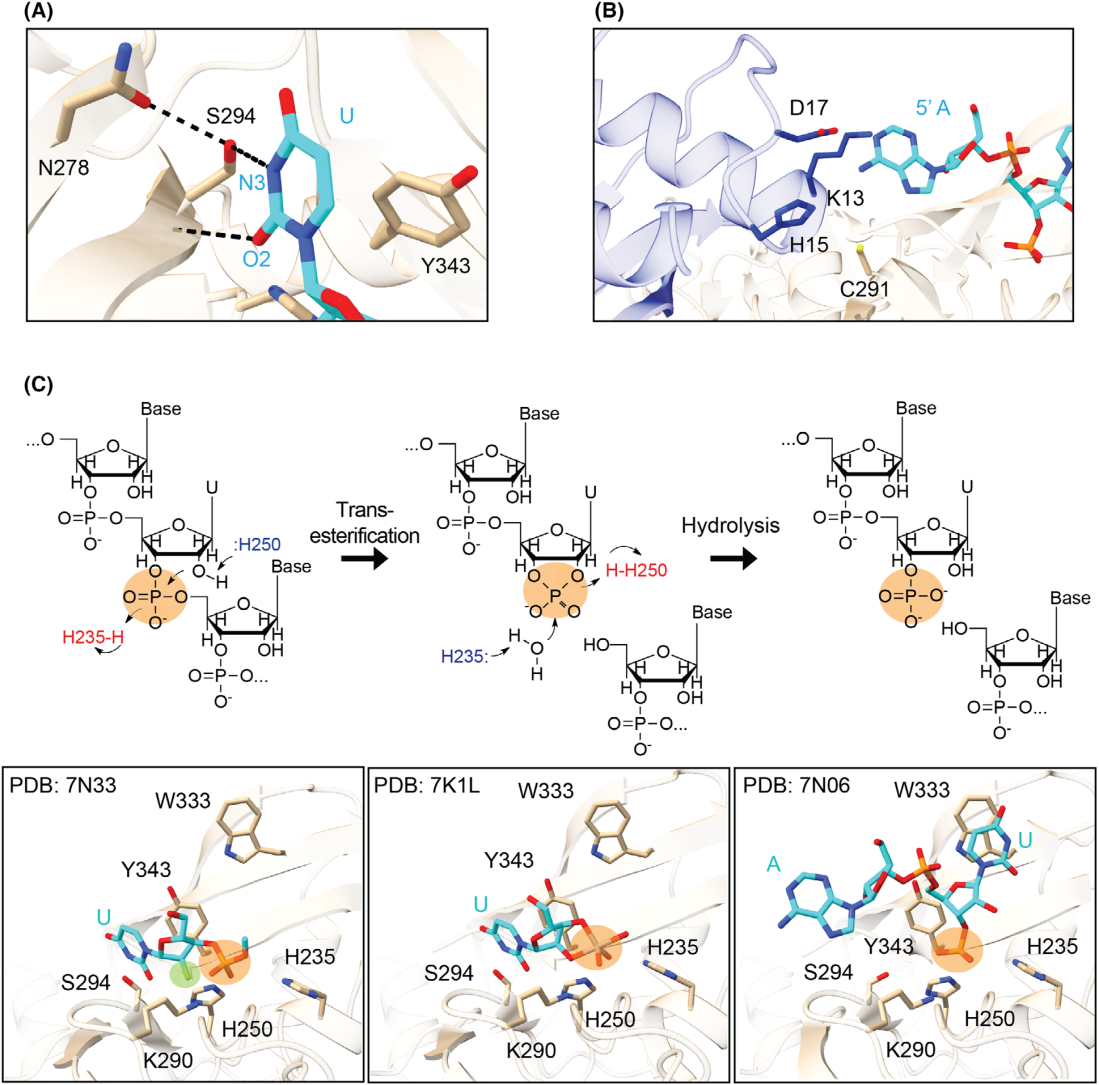
Nsp15 endoribonuclease cleavage mechanism. (A) Uridine discrimination occurs via a specific hydrogen bond network; N278 positions S294 to form a hydrogen bond with the N3 position of uridine. Y343 supports the position of the base through van der Waals interactions with the ribose sugar (PDB: 7N33). (B) A postcleavage structure showed interactions between the 5′ base and N‐terminal residues of a neighboring protomer (shown as blue sticks), mediated by water (PDB: 7N06). (C) The two‐step metal ion‐independent cleavage reaction shown in schematic form (top), with active site snapshots from structures of each state below (PDB: 7N33, 7K1L, 7N06) [49, 66]. Bottom: Nsp15 residues (tan) and RNA (cyan) are shown in stick format. The scissile phosphate is highlighted with an orange circle in both views. Prior to cleavage, the uridine is positioned for cleavage, interacting with the discriminatory residue S294. The position highlighted in green in the bottom left panel is the 2′ fluorine modification to prevent cleavage, showing the 2′ position is positioned in the catalytic triad for cleavage. Following the transesterification reaction, the uridine base and cyclic phosphate are positioned in the same places (central panel). After hydrolysis, however, the uridine base pivots to π‐stack with W333 (right panel).

Numerous X‐ray crystallography and cryo‐electron microscopy cryo‐EM structures of the Nsp15 EndoU domain have been determined, with good RMSD agreement (Table 2) [49, 66]. The EndoU domain resides in the C‐terminus of Nsp15 in CoVs and is comprised of a conserved EndoU fold (Fig. 4). In alpha‐ and beta‐CoVs, including SARS‐CoV‐2, Nsp15 forms a hexamer of back‐to‐back trimers, such that the EndoU domain faces outwards [48, 50, 51, 53, 67]. The EndoU domain is a small, mixed fold with alpha helices and beta sheets. Five alpha helices form a bundle that sites on top of two sets of three‐stranded, antiparallel beta sheets. As in the well‐studied endoribonuclease RNase A, the EndoU catalytic triad is composed of two histidines (H235 and H250 in SARS‐CoV‐2) and one lysine (K290 in SARS‐CoV‐2). The active site sits in a groove formed by the two β‐sheets and the edge of the helical bundle and includes residues involved in interacting with the scissile phosphate (H235, H250, K290), the uridine nucleotide (Y343, S294), and bases 5′ and 3′ the uridine (W333; [48, 49, 50]). The N‐terminal domain of a neighboring protomer also forms part of the distal active site, through water‐mediated contacts with the 5′ base of ssRNA, pointing to a reason Nsp15 must oligomerize to function (Fig. 5) [66].

**Table 2:** PDB IDs for published coronavirus Nsp15 structures. PDBs of published coronavirus Nsp15 structures are listed alphabetically by PDB ID.

PDB ID	Species	Protein components	State/ligands	Structural technique	Reference
2GTH	MHV	WT Nsp15		X‐ray diffraction	[52]
2GTI	MHV	Nsp15 F307L		X‐ray diffraction	[52]
2H85	SARS‐CoV‐1	WT Nsp15		X‐ray diffraction	[96]
2OZK	SARS‐CoV‐1	N‐terminal truncated Nsp15		X‐ray diffraction	[51]
2RHB	SARS‐CoV‐1	Nsp15 H234A		X‐ray diffraction	[50]
5YVD	MERS	WT Nsp15		X‐ray diffraction	[69]
6VWW	SARS‐CoV‐2	WT Nsp15		X‐ray diffraction	[67]
6W01	SARS‐CoV‐2	WT Nsp15	Citrate	X‐ray diffraction	[67]
6WLC	SARS‐CoV‐2	WT Nsp15	Uridine‐5′‐monophosphate	X‐ray diffraction	[49]
6WXC	SARS‐CoV‐2	WT Nsp15	Tipiracil inhibitor	X‐ray diffraction	[49]
6X1B	SARS‐CoV‐2	WT Nsp15	GpU	X‐ray diffraction	[49]
6X4I	SARS‐CoV‐2	WT Nsp15	3′‐uridine monophosphate	X‐ray diffraction	[49]
7K0R	SARS‐CoV‐2	WT Nsp15	Uridine‐5′‐triphosphate	Cryo‐EM	[48]
7K1L	SARS‐CoV‐2	WT Nsp15	Intermediate mimic; Uridine‐2′,3′‐vanadate	X‐ray diffraction	[49]
7N06	SARS‐CoV‐2	WT Nsp15	Post‐cleavage state; AU‐ 3′ monophosphate	Cryo‐EM	[66]
7N33	SARS‐CoV‐2	WT Nsp15	Pre‐cleavage state; AUA	Cryo‐EM	[66]

### Nsp15 cleavage mechanism

Nsp15 primarily cleaves 3′ of uridines using a metal ion‐independent mechanism, although it has some activity towards cytidines [50]. Structures with nucleotide mimics or short RNA oligos show the base recognition site is not large enough to fit purines [48, 49]. *In vitro* nuclease assays have demonstrated a requirement for Mn^2+^ for optimal activity; Mg^2+^ and other divalent cations support low levels of activity [68, 69]. However, the concentration used in *in vitro* assays is greater than *in vivo* concentrations, and it is unknown whether Mn^2+^ plays a role in infected cells. Furthermore, none of the available structures have identified the location of any Mn^2+^ ions.

Nsp15 uses the same mechanism as RNase A: a two‐step transesterification reaction whereby the roles of the two histidine residues switch following the first step [48, 70, 71]. Briefly, H250 acts as a general base and abstracts a proton from the 2′‐hydroxyl of the uridine. This leads to an attack on the scissile phosphate, generating a 2′–3′‐cyclic phosphate on the uridine, while the 3′ base leaves with a 5′‐hydroxyl group. In the second step, H235 acts as a base to activate a water molecule, which attacks the cyclic product to yield a 3′‐monophosphate. Newly determined structures now represent the major steps in the cleavage reaction: pre‐cleavage, post‐transesterification (2′–3′‐cyclic intermediate), and post‐hydrolysis (Fig. 5). While this two‐step reaction is very efficient for RNase A, we previously demonstrated that Nsp15 has a suboptimal position of the second histidine (H235), which results in a greater yield of the cyclic phosphate product due to a slower hydrolysis step [66]. Nsp15 cleavage has largely been studied on ssRNA [48, 68, 69]; however, CoV RNA forms secondary structures, and transitions through a double‐stranded dsRNA during replication. A recent computational model [22] proposed that Nsp15 serves as a scaffold for the RTC, placing it in close contact with dsRNA. It remains unclear how Nsp15 processes dsRNA, as base‐paired RNA would have to undergo a conformational rearrangement to fit into the Nsp15 active site.

### Nsp15 substrate specificity

Nsp15 has broad specificity towards uridine‐containing RNA substrates. A specific hydrogen bond network in the active site between the O2 and N3 atoms of the uridine and SARS‐CoV‐2 Nsp15 residues S294 and N278 successfully discriminate uridine in ssRNA (Fig. 5) [48, 49, 66]. These residues are also conserved in SARS‐CoV‐1 and MERS Nsp15, which leads to conserved substrate specificity [50, 69]. Thus, substrate specificity for Nsp15 is predominantly driven by interactions with the uridine base, which positions the scissile phosphate for cleavage (see above). However, biochemical analyses of the model MHV CoV revealed some cleavage 3′ of cytidines as well [50, 72]. The RNA sequencing analysis did not reveal an extended motif Nsp15 recognizes for cleavage but did show that the base 3′ of the uridine was often adenine [72]. Biochemical analyses characterizing SARS‐CoV‐2 Nsp15 similarly revealed a preference for a purine 3′ of the uridine, but no preference 5′ to the uridine [66]. Cryo‐EM structures further revealed that ssRNA was not fixed in place and did not have extensive contact with the protein; density was resolved for only the 5′ or 3′ bases surrounding the uridine [66]. Altogether, Nsp15 appears to broadly cleave ssRNA due to a lack of extended substrate recognition surface and the primary interactions being with the uridine nucleotide.

While the action of Nsp15 on ssRNA has been well‐studied, the substrate specificity of dsRNA has not been well‐characterized. Early work with SARS‐CoV‐1 and HCoV‐229E (Human CoV‐229E) on dsRNA found preferential cleavage after uridines with purines downstream, very similar to the preferences noted above [73]. Analyses of SARS‐CoV‐1 showed Nsp15 prefers to cleave unpaired uridines within duplex RNA, suggesting secondary structure could have a large impact on RNA cleavage activity [74]. *In vitro* work has provided many of the mechanistic details about cleavage and substrate specificity but cannot answer whether Nsp15 distinguishes between the positive and negative strands. In recent work by Ancar et al. [72], they employed a probe that specifically captured 2′–3′‐cyclic phosphates to isolate Nsp15‐specific cleavage products in MHV. Their analyses found cleavage sites throughout the positive strand of the genome but did not detect negative‐strand products. However, previous work with MHV had found the negative strand was predominantly affected by Nsp15 cleavage, with Nsp15 primarily targeting the poly‐U tail at the 5′ end of the negative strand [62]. Poly‐U sequences are known PAMPs, therefore this finding provides a definitive link between Nsp15 substrates and immune evasion. Given that Nsp15 does not have specificity beyond uridines, there is likely additional regulation *in vivo* to ensure positive‐strand replication and packaging in new virions without cleavage. Interactions with other nsps or spatial separation in DMVS or other rearranged membrane compartments may also regulate Nsp15.

## Nsp14 and Nsp15 and the replication transcription complex

While there are no higher order complexes of the RTC SARS‐Cov‐1 outside of the core (Nsp12, 7, 8) [75], recent work has captured structures of the RTC of SARS‐CoV‐2 with additional nsps [5, 15, 17, 19]. To date, however, there has been no RTC structure with Nsp15 determined experimentally, suggesting it may only associate with the RTC transiently. Previous work with *in situ* tagged Nsp15 in MHV found RNA‐independent interactions with Nsp8 and Nsp12 [20].

How Nsp14, together with its cofactor Nsp10, interacts with the RTC to carry out both of its functions is better understood. The Nsp14/10 complex interacts with the core RTC (Nsp12, 7, 8), in addition to other Nsps to provide a proofreading function during replication. The Nsp14/10 complex also interacts with the RTC and Nsp9 as an intermediate step in mRNA capping, forming a “Cap (0)‐RTC complex” (PDB ID: 7EIZ), whereby Nsp9 and Nsp12 stabilize the association of Nsp10/Nsp14 ExoN in the complex [19]. When the RTC misincorporates a ribonucleotide, the replication machinery stalls, preempting the removal of the mispair through the exonuclease activity of Nsp14/10 before RNA polymerization restarts [46]. How Nsp14 accesses the mismatch end after incorporation is still under investigation, but recent structural analyses have revealed possible mechanisms, including backtracking and polymerase (Nsp12) release. Backtracking is a common mechanism employed by DNA‐ and RNA‐dependent polymerase during proofreading. In the context of the active replication, Nsp14 does not directly contact RNA [19], and therefore, there is likely a large conformational change or association and dissociation of protein factors that occur prior to proofreading and/or during “backtracking”. Recent evidence shows the SARS‐CoV2 helicase, Nsp13, may engage the RNA and promote movement of the RTC machinery in the reverse direction to “backtrack” [15]. The action of Nsp13 could either allow direct access to the mismatch or induce the subsequent release of Nsp12 revealing the mismatch. A role of Nsp14 in backtracking has been suggested from a dimeric “Cap (0)‐RTC complex” which describes not only the interaction of Nsp14 with Nsp9, and RTC, but also a C‐terminal interaction with nsp13 (PDB ID: 7EGQ [19]). Nsp8 has been shown to contact Nsp14 bound to an RNA mismatch (PDB ID: 7NOC, [36]), given that Nsp8 is a cofactor for Nsp12, perhaps its presence in this complex supports a theory of Nsp12 release to reveal the mismatch [36]. The intermediary step of mRNA capping has also revealed protein‐to‐protein interactions with Nsp12 and Nsp8 [19].

## Inhibitors of Nsp14 and Nsp15

The safe, effective vaccines developed against the SARS‐CoV‐2 spike protein changed the course of the COVID‐19 pandemic. However, the virus continues to spread in communities and has led to variants with varying degrees of vaccine evasion. This highlights the need to develop antiviral treatments for SARS‐CoV‐2; the enzymatic nsps are attractive candidates due to their roles in promoting virus survival, through replication or immune evasion.

Due to its important functions in the coronavirus life cycle, and the fact that its bifunctionality permits two potential sites of inhibition, Nsp14 is an attractive antiviral target. To date, the majority of inhibitors designed against Nsp14 have focused on the C‐terminal domain, specifically MTase and SAM‐dependent inhibition (reviewed in Ref. [16]). In addition to the C‐terminal region, the ExoN and regions of protein–protein interactions are also potential targets for drug design. In general, it can be speculated that the lack of sequence overlap of the active site of ExoN (i.e., D90, E92, E191, D273, and H268 residues) with the human proteome, combined with the structural and sequence similarity in the ExoN/Nsp10 protein interface across coronaviruses may further warrant investigation of these sites as inhibitor targets [39].

Rapidly advancing research has begun to uncover a variety of potential ExoN active site inhibitors (e.g., glucocorticoids, ritonavir [76], repurposed antivirals [77]). Nsp14 ExoN could also be inhibited using chemically modified nucleoside analogs, which will either evade proofreading of Nsp14 and/or inhibit Nsp12. One inhibitor in this class, which has been approved for the treatment of COVID‐19, is remdesivir, a nucleoside analog with a 1′‐cyano group. This modification allows for chain incorporation by the RTC, then inhibition through delayed chain termination and stalling [17]. The 1′‐cyano group can also disrupt the cleavage site of ExoN via steric interactions and lead to a further reduction in the cleavage efficiency. Unfortunately, mutations in Nsp14 lead to inhibition escape and proofreading allows for resistance to this pathway of inhibition [35, 78, 79].

Recent research efforts have also led to the design of medium‐ and high‐throughput inhibitor assays for studying the inhibition of Nsp14 ExoN [80, 81]. These assays (FRET‐based) provide a quick throughput and have already shown promise to identify predicted active site inhibitors with low micromolar (µm) IC_50_ and decreased EC_50_ in combination with remdesivir. It remains to be seen how uptake or patient tolerance would respond to inhibitors that have shown promise in *in vitro* conditions. It also seems attractive to repurpose drugs; however, repurposing current antiviral inhibitors generally requires higher dosages for inhibition as indicated by µm range IC_50_ [78]. In all, new structural analyses combined with efforts to develop high‐throughput assays will undoubtedly promote future structure‐guided inhibition studies for Nsp14 ExoN inhibition.

Given that Nsp15 is a promising target for the development of new antivirals, there have been several *in silico* docking and high‐throughput screening (HTS) methods developed over the past two years to identify Nsp15 inhibitors. Nsp15 is suitable for HTS methods because cleavage activity can be robustly measured *in vitro* using small fluorescently labeled RNA substrates containing a single uridine cleavage site [66, 69, 82, 83]. A screen of a 5000‐compound library identified NSC95397, which is a known inhibitor of Cdc25 phosphatase and several kinases, as a specific inhibitor of Nsp15 activity *in vitro* [84]. While this inhibitor was not effective at inhibiting SARS‐CoV‐1 viral growth in Vero cells and showed some toxicity issues, it could be a starting point for future drug development [84]. A similar HTS of over 13 000 compounds similarly identified several small molecules that inhibit Nsp15 *in vitro* and could be further optimized [83]. In addition to *in vitro* HTS approaches, cell‐based screens have also identified putative Nsp15 inhibitors. A cell‐based screen of derivatives of a 1,2,3‐triazole ring revealed a compound that prevented the formation of dsRNA intermediates in HCoV‐299E infected cells and molecular modeling suggests that this inhibitor binds near the catalytic center of Nsp15 [85].

High‐resolution structures of SARS‐CoV‐2 Nsp15 also provide a platform for future drug development [49, 66, 67]. For example, the uridine‐binding pocket within the Nsp15 active site is a promising drug target. Indeed, a crystal structure of SARS‐CoV‐2 Nsp15 bound to Tipiracil, a uridine analog and FDA‐approved drug for colorectal cancer, revealed Tipiracil bound in the uridine pocket, suggesting that it is a competitive inhibitor of Nsp15 [49]. Tipiracil was further shown to inhibit Nsp15 nuclease activity *in vitro*; however, it only displayed modest viral replication inhibition in whole‐cell assays [49]. While Tipiracil alone does not appear to be suitable as an antiviral, exploration of other uridine derivatives is worth investigating in future. Another recent study identified additional FDA‐approved drugs that can inhibit Nsp15 activity *in vitro* [86]. Using high‐resolution structures as the starting point, *in silico* screening approaches have also identified numerous putative Nsp15 inhibitors [87, 88, 89, 90, 91, 92].

## Summary and open questions

Structures of new states and more detailed substrate analyses have provided insight into the actions of the coronavirus nucleases, Nsp14 and Nsp15, but there are still many outstanding questions. While it is well‐characterized that Nsp10 interacts with the ExoN domain of Nsp14 to stimulate the 3′–5′ exonuclease activity of Nsp14, the mechanism of stimulation is unclear. Nsp10 likely stimulates through active site stabilization or RNA‐binding contribution [35] but more research is needed. Furthermore, an allosteric interaction cannot be ruled out, yet there is no evidence of this in structural studies. The Nsp14/10 exonuclease reaction mechanism is clearly metal‐dependent, but defining whether the reaction proceeds via one‐ or two‐metal reaction mechanism would provide further insight into the specificity of Nsp14/10. There is new structural evidence unveiling the protein interface of Nsp14 with the RTC, but how and if Nsp14 regulates the activity of this complex or how the complex may change Nsp14 exonuclease activity is unknown. Nsp10 is a cofactor for both the capping and exonuclease activity of Nsp14, yet Nsp10 is also critical for Nsp16 activity [93, 94] using nearly the same interaction interface. The transfer of Nsp10 as the presumable limiting factor in capping is still under investigation. Likewise, it is unknown whether there is a change in the Nsp10/14 interface or whether Nsp14:RNA interactions are altered in the presence of different substrates or during backtracking. A thorough understanding of this interface could provide a framework for future inhibitor design as a means to regulate Nsp14 activity.

Structures from each step of the Nsp15 reaction mechanism [49, 66] illustrate how Nsp15 recognizes uridine and carries out cleavage. However, there is still no structural evidence for the stimulating role of Mn^2+^. Since Nsp15 cleaves in a metal ion‐independent manner, the role of Mn^2+^ is likely to charge stabilization or orientation. Biochemical assays with Nsp15 have shown it can cleave both ss and dsRNA. Work remains to characterize dsRNA preferences *in vitro* and identify the preferred substrate *in vivo*. Studies so far have shown that Nsp15 lacks specificity beyond uridine recognition, raising questions about how EndoU activity is regulated in infected cells. The coronavirus genome must successfully replicate to be packaged in virions, so Nsp15 is unlikely indiscriminately cleaving all viral RNA. Is there a mechanism for specific cleavage of the negative‐strand RNA, which serves as a PAMP? Molecular dynamics simulations predict Nsp15 interactions with Nsp14 [22]. It is hypothesized that Nsp14 ExoN could bridge an interaction between Nsp12 and Nsp15 to facilitate replication processivity; however, previous two‐yeast hybrid analyses for SARS‐CoV‐1 do not show evidence of this interaction [22, 95]. It remains to be seen how Nsp15 interacts with the rest of the RTC, and with that, how Nsp15 is regulated. Experimental validation also awaits the many *in silico* Nsp15 inhibitor screens that have been carried out. Increasing understanding of the cleavage mechanism and substrate specificity of Nsp14 and Nsp15 through structural biology and biochemistry will allow for more rational drug design to develop antivirals against SARS‐CoV‐2.

## Conflict of interest

The authors declare no conflict of interest.

## Author contributions

MNF, AAR, IMW, WCC, and RES conceived and wrote the manuscript.

## Data Availability

No new data was generated in this review.
